# An intelligent methodology for the use of multi-criteria decision analysis in impact assessment: the case of real-world offshore construction

**DOI:** 10.1038/s41598-022-19554-1

**Published:** 2022-09-07

**Authors:** Mariza Tsakalerou, Damianos Efthymiadis, Almat Abilez

**Affiliations:** 1 Department of Civil Engineering, Nazarbayev University, 53 Kabanbay Batyr, 010000 Nur-sultan, Kazakhstan; 2 SemArco LLP (Archirodon Group), 52/1 Mangilik El, 010000 Nur-sultan, Kazakhstan

**Keywords:** Civil engineering, Socioeconomic scenarios

## Abstract

Impact assessment of large-scale projects involves a plethora of technical, economic, social, and environmental factors that must be assessed along with the expectations of the stakeholders of each project. While impact assessment is required for a development project to receive regulatory approval to proceed, it is also an invaluable tool during the design phase of complex projects, providing for informed decision-making. Molding multiple perspectives of diverse stakeholders into a single collective choice is a key challenge in the process. Multi-Criteria Decision Analysis (MCDA) is the methodology used to rank a finite number of decision options based on a finite set of evaluation criteria. Different MCDA techniques, however, may lead to different decisions when applied to the same problem while different sets of criteria and weights may rank choices differently even when the same method is applied. This is a cause of concern, and even acrimony, amongst the stakeholders, often leading to protracted periods of negotiation and delaying project launching. The objective of this paper is to present an intelligent system to ameliorate the effects of the inherent subjectivity in MCDA techniques and to develop a consensus amongst the stakeholders in a data-driven setting. A case study from the field of offshore construction is used as a running example. This case study, informed by real-world experience in the field, demonstrates succinctly the issues involved and illustrates clearly the proposed intelligent methodology and its merits.

## Introduction

Impact assessment is a formal, evidence-based prospective analysis to estimate the attributable impact of a project and to inform decision makers accordingly. This *ex-ante* impact analysis is a part of the planning stage of any development project and often leads to repeated corrective cycles in the design process. (*Ex-post* impact assessment, measuring the actual impact after the completion of a project, is beyond the scope of this paper.) The focus of impact assessment in the past was rather narrow, examining a tightly defined set of—mostly economic-indicators. Impact assessment today, though, is subject to regulatory oversight in the OECD countries and its focus evolved tremendously to cover environmental, social, sustainability, as well as economic effects of large-scale projects. The impact assessment process often provides opportunities and mandates roles for public engagement, reconciliation, and partnership in the public interest^1^.

There are of course several methodological challenges to impact assessment. For instance, identifying and predicting the environmental, health, social and economic impacts of design choices is extremely difficult, especially since the anticipated effects may occur in different time frames. Aggregating or comparing several aspects of impact, measured across diverse scales, is also difficult due to potential complex interactions between them. Extended time horizons require assumptions about societal and technological changes that may occur over the expected lifetime of a project. Ultimately, impact assessment is not neutral, often causing Hawthorne effects whereby monitoring the impact of a process leads to improvements in it due to the awareness of being observed.

Impact assessment is currently a mandatory ingredient of development project portfolios requesting regulatory approval. Beyond its regulatory significance, impact assessment is an invaluable tool during the design phase of large complex projects, providing for informed decision-making^2^. Indeed, impact assessment is credited with: (i) increased participation in the decision-making, reflecting a wide range of stakeholder considerations while making more difficult the pursuit of special interests; and (ii) improved transparency in the decision-making by disclosing to the stakeholders the anticipated impact of specific design choices during the design phase. The open and transparent process of impact assessment is of course not without its own pitfalls. Decision analysis tools may often rank alternatives differently based on initial assumptions and choices. Decisions and decision-making are, however, understood in diverse ways by different stakeholders and decision makers may therefore act differently when faced with diverse or even conflicting results^3,4^.

In this broader context, the objective of this paper is not to invent a new decision analysis technique but to present a novel intelligent methodology to ameliorate these real-world problems using existing methods in the decision analysis toolbox. While this methodology is applicable to a broad range of development projects, this paper utilizes a representative case study of real-world offshore construction as a running example to demonstrate succinctly the issues involved and illustrate clearly the proposed intelligent methodology and its merits.

Offshore construction is the installation of structures and facilities for a variety of functions in a marine environment. Shore and harbor protection facilities, offshore oil & gas drilling platforms, underwater pipelines, floating solar farms, offshore wind farms, are but a few examples of offshore installations. Offshore structures are constructed worldwide primarily for the energy sector (mostly oil & gas but, increasingly, solar and wind) and have significant economic impact for the regions and firms involved^5^.

An offshore installation is typically a complex and expensive engineering structure composed of many subsystems. Each installation is usually unique with its own design adapted to specific operational characteristics such as water depths, wave patterns and overall environmental conditions. Offshore construction is characterized by an intricate quagmire of technical, economic, and regulatory factors that affect the financial viability, the designed lifetime, and the management of expectations of the stakeholders of each project^6–8^. With construction taking place in increasingly exacting locations, disputes arising from cost overruns, scheduling delays and technical difficulties frequently end up in litigation^9^.

Firms involved in offshore construction must address decision-making problems at every step of the process, to support the choices of those who are making the decision. Decision-making in this context involves the comparative analysis of a finite set of alternatives described in terms of a finite set of evaluation criteria. The molding of multiple perspectives from different stakeholders into a single collective choice is based on the values and preferences of the decision makers who are not necessarily (or exclusively) of the construction firm. Choosing an alternative amidst conflicting multiple criteria and multiple perspectives of stakeholders is a challenging task. In such complex situations, multi-criteria analysis is often used as an *ex-ante* evaluation tool to rank a finite number of decision options based on a finite set of evaluation criteria^10–12^.

The term Multi-Criteria Decision Analysis (MCDA) encompasses the wide-ranging family of systematic and transparent methods providing for the rational differentiating between a range of options, based on a set of criteria, against which each option is assessed. A typical MCDA method evolves along five structured stages: (i) defining the decision problem, goal, or objective; (ii) determining the criteria and the constraints; (iii) assessing the importance of the criteria with respect to each other and the objective; (iv) aggregating all the information into a common value metric; and finally (v) ranking the alternative choices. While several MCDA methods may be applicable to a decision-making situation, there are no clear guidelines on how to choose the most appropriate one. The choice of the MCDA method can significantly affect the quality of the decision, as different methods may lead to different decisions when applied to the same problem. More importantly, different sets of criteria and weights may rank choices differently even when the same method is applied.

Decision-making problems in offshore construction are often defined by multiple objective profiles as well as by multiple success factors needing to be evaluated. During the distinct phases of the construction process, decision-making methodologies must be employed to interpret the subjective preferences of the stakeholders and to translate them into real actions. MCDA techniques do support the management of multiple factors in the quest for an optimized decision, and their use is an established practice in offshore construction.

In offshore construction it is critical to assess not only the technical and economic factors but also the social and environmental impact of choices at the design stage^13,14^. Indeed, offshore installations raise a morass of policy assessment issues stretching well-beyond beyond market impact. Reflecting the value of the natural environment and the wellbeing of the communities affected in design decisions is essential for the sustainable development of offshore installations^15,16^.

Visual and landscape impact on tourist activities, archaeological concerns, chemical and noise pollution, safety concerns from sabotage and terrorism, biodiversity risks etc. are essential elements of the offshore construction process. While MCDA techniques can in principle account for such elements, many of these factors are difficult to measure and the impact of design choices may play out over extended periods of time. Often the use of non-monetary evidence provides a more meaningful picture, leading to mixed models with quantitative and qualitative criteria on the same canvas. In real-world problems, MCDA methods are challenged to integrate into a coherent framework the missing or imprecise data, the heterogeneous information, and the extended intervention horizons present in offshore construction^17,18^.

From the simple to the highly sophisticated, MCDA methods incorporate into the analysis the subjective judgement of stakeholders, that is the individual or entities who are directly or indirectly affected by the outcome of a decision-making process. In offshore construction, the stakeholder group includes the expected range of technical and financial specialists but also representatives of political, administrative, and regulatory authorities and public, private, and community organizations. Stakeholder judgement is important in selecting the relevant criteria, in identifying their weights, and in scoring the options examined against each objective. While this approach is at the core of MCDA techniques, its subjective nature is often a cause of concern, and even acrimony, amongst the stakeholders, often leading to protracted periods of negotiation^19,20^.

One way to address the issue of subjectivity in MCDA methods is to use more robust hybrid models that combine two or more techniques to address decision-making problems. The expectation from a hybrid approach is that it will combine the advantages of each MCDA method while overcoming the drawbacks of each method applied alone. Hybrid MCDA methods can also effectively support the structuring of decision making on complex policy issues with fuzzy data and simultaneous use of quantitative and qualitative variables^21,22^.

Coupling MCDA techniques is often done within the framework of designing intelligent Decision Support Systems (IDSS). Such systems have been shown to have considerable success in addressing a wide range of complex real-world problems, at the expense of course of the level of transparency apparent to external stakeholders. In this context, the objective of this paper is threefold:

- To elucidate the view from the field on the use of MCDA techniques in offshore construction;
- To detail some of the most persistent practical issues in the use of existing MDCA methods; and
- To present an IDSS employing existing MCDA tools for the use of firms involved in the offshore construction of marine installations.

While the case study is informed by real-world experience in the field of offshore construction, the novel IDSS methodology-which is the key contribution of this paper- is applicable across a wide range of development projects facing similar issues.

This paper is organized as follows. In “MCDA—the view from the field” Section, a concise overview of MCDA techniques in offshore construction practice is presented. The overview is based primarily upon the experiences and views of the second author, who is the General Director of Archirodon Group NV-one of the top marine contractors internationally with 60 years of experience in offshore construction. In “Offshore wind farm installation—a case study” Section, a case study from the literature is employed to demonstrate practical issues with MCDA techniques such as the infamous rank-reversal and how they may affect stakeholder perceptions. In “The rank reversal conundrum and identifying the top choices” Section, an intelligent DSS is presented that couples a traditional MCDA approach with fuzzy sets theory to ameliorate the issues identified in the previous section. Finally, in “Fuzzy logic and criteria clustering” Section, the conclusions of this paper and some directions for future research are presented in summary form.

## MCDA—the view from the field

The appeal of MCDA in many real-world applications is due to its capacity to simplify complex situations characterized by multiple (and possibly conflicting) objectives and criteria, and to rationalize the decision process. The common schema of MCDA typically involves construction of a performance matrix, with each row representing a specific decision option and each column assessing the performance of that option against each of the criteria set. MCDA involves two critical choices: (i) the selection of criteria that capture the most important parameters, constraints and expected impacts of a project; and (ii) the weighting of the criteria to reflect their relative importance^23,24^.

In real-world situations, such as offshore construction, where the selection of the criteria is not always obvious, and the data is often fuzzy, significant human resources are devoted to the structuring of the problem. Identifying and selecting the individuals that will be involved in the analysis is a process critical for success, yet rife with technical, political, and human relations issues. The project contractor is typically responsible for appointing -after consultations- three teams to be involved in the application of MCDA:

- The negotiation team, with members chosen among the project’s stakeholders and whose preferences and ratings will inform the structure and entries of the MCDA performance matrix;
- The technical team responsible for supporting the judgement team group that includes members proficient in the mathematics of MCDA and the relevant software implementations as well as experts responsible for providing additional data to the negotiation group as needed; and
- The mediation team with managerial and legal expertise to safeguard the fairness of the process and resolve arguments.

The negotiation team, with the tacit support of the technical team, proceeds sequentially to establish: (i) the list of potential decisions or solutions to be examined in the analysis; (ii) the criteria to be used by integrating all the points of view expressed; (iii) the relative importance of each criterion; (iv) the rating of each solution when judged against each criterion; and (v) the aggregate judgements using an agreed upon MCDA technique. The mediation team makes sure that each step of the process unfolds within a framework established a priori, with rules agreed by all, so that the process will result in decisions with the broadest possible acceptance.

In practice, the process outlined previously rarely concludes in one round. Typically, there are several iterations that may modify the definition of the problem, the criteria used, and the assessments made. Revisiting the criteria and rating their importance is a useful negotiation tool for debates among the contractor and the stakeholders. These iterations test the boundaries of the decision (and may even serve as a de-facto sensitivity analysis) until the proposed solution meets with general acceptance.

What is rarely appreciated in theoretical MCDA is the fact that the analysis (with or without iterations) takes considerable time. In offshore construction projects, for instance, it usually lasts several months. The distinct danger in such time spans is that some of the fundamental economic, social, or political dimensions may *indeed* change due to external factors, lengthening even further the decision horizon. In the experience of major international offshore contractors such as Archirodon, slow decision making -and the resultant design changes- is the top factor for cost overruns. The Archirodon experience, is not unique; a comprehensive analysis of risk factors facing construction management firms cites the lack of robust risk management practices as a distinct threat to profitability, project performance, and customer satisfaction^25^.

In such complex situations where there is a need to reach a timely decision and time is of essence, the MCDA methodology should be as simple as possible, and the dimensions of the performance matrix kept to a minimum. That is, the choices compared should be as few as it is realistic, while at the same time the criteria used should be few and easily understood by the stakeholders. Furthermore, experienced contractors make sure that there is real participation and deliberation in the application of MCDA to reduce unnecessary iterations. In this context, participation extends beyond information dissemination to include active engagement and exchange of ideas. Deliberation involves fair and inclusive dialogue between participants able to debate and contribute to the methodology.

MCDA presents a shared framework and a common language to develop data-driven solutions for complex offshore installations but is particularly sensitive to subjective biases and data asymmetry. Participation and deliberation are essential for a timely decision, yet they are accompanied by problems of their own. Practical difficulties arise when:

- The stakeholders do not have basic skills in mathematical concepts and data aggregation methodologies to appreciate the nuances in MCDA; or
- The stakeholders do have the skills to understand the subjectivity inherent in MCDA, leading to fears that the manipulation of criteria and weights may privilege certain choices over others.

In the sequence, a running case study is employed to highlight some of the issues involved. To avoid using proprietary information from Archirodon, and to alleviate possible concerns about a conveniently designed example, the case study is based on publicly available data for a specific problem of designing an offshore solar farm installation^26^. All data generated or analyzed during this study are included in the body of this paper.

## Offshore wind farm installation—a case study

The case study involves the problem of site selection of an offshore solar farm deployment in the Aegean Sea, Greece^26^. There were nine candidate locations (MA1 ÷ MA9) and seven assessment criteria (AC1 ÷ AC7) as outlined in Table 1. The nine locations have been chosen from a larger pool of choices after the application of exclusion criteria and the removal of unsuitable areas. The assessment criteria were identified through a literature review of renewable energy sources to include water depth (AC1), distance from shore (AC2), main voltage at a maximum distance of 100 km from the site area (AC3), distance from ports (AC4), serving population (AC5), solar radiation (AC6), and installation site area (AC7). AC1, AC2 and AC4 have *negative* polarity (smaller is better) while AC3, AC5, AC6, and AC7 have *positive* polarity (larger is better).

**Table 1:** Initial assessment matrix.

	AC1 (m)	AC2 (km)	AC3 (kV)	AC4 (km)	AC5 (Population)	AC6 (kWh/m^2^)	AC7 (km^2^)
MA1	100	11–25	150	≤ 50	686 969	1801–1900	0.973
MA2	100	11–25	150	≤ 50	119 887	1801–1900	1.071
MA3	100	11–25	150	≤ 50	176 264	1701–1800	1.112
MA4	100	11–25	66	51–70	176 264	1801–1900	1.322
MA5	100	26–50	66	≤ 50	176 264	1701–1800	4.885
MA6	100	26–50	66	51–70	176 264	1601–1700	1.669
MA7	50	11–25	66	51–70	176 264	1601–1700	0.974
MA8	50	11–25	400	≤ 50	176 264	1601–1700	1.615
MA9	50	11–25	400	≤ 50	176 264	1601–1700	3.628
Polarity	Negative	Negative	Positive	Negative	Positive	Positive	Positive

Since the criteria are expressed in truly diverse scales and units, it is customary to proceed with *normalization*, to make all the indicators comparable on the same scale, and *aggregation,* to combine the normalized indicators in an overall score/index. For MDCA input data, there are varied techniques of normalization (ordinal, linear scale, ratio scale, sigmoid etc.) and aggregation (additive, geometric, harmonic, minimum, median etc.). The actual combination of normalization and aggregation method used influences the outcome of MCDA.

For the present case study, the web-based MCDA Index Tool (www.mcdaindex.net) is used to further analysis. The MCDA Index Tool provides for all combinations of 8 normalization methods (rank, percentile rank, standardized, minmax, target, logistic, 3-tier categorical, 5-tier categorical) and 5 aggregation methods (additive, geometric, harmonic, minimum, median). Since not all normalization methods are compatible with all aggregation methods, there are 31 feasible combinations of normalization/aggregation^27^.

**Figure 1:**
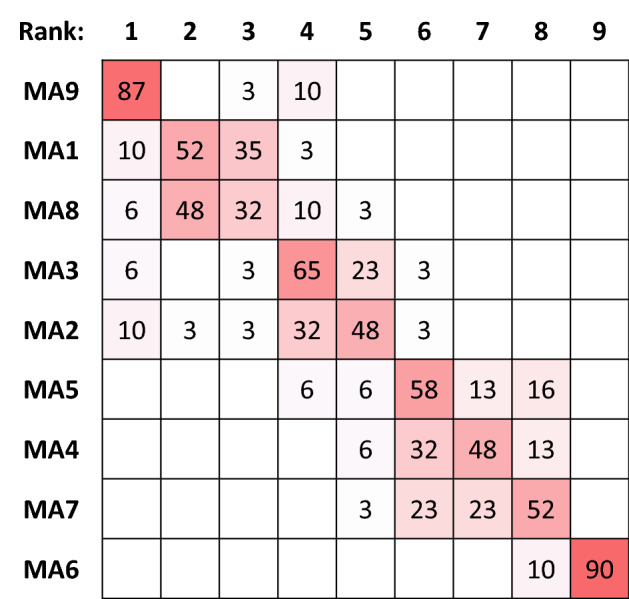
Ranking Strength over all normalization/aggregation combinations.

Processing the input data of the case study with the MCDA Index tool for 31 distinct combinations of normalization and aggregation methods with equal weights leads to the results tabulated in Fig. 1. The color coding in the figure shows the strength of the ranking obtained by each alternative location. For instance, location MA9 is top-ranked in 87%, third-ranked in 3% and fourth-ranked in 10% of the 31 normalization/aggregation pairs examined. Figure 1 illustrates the alternative rankings that can be obtained for different pairs.

**Figure 2:**
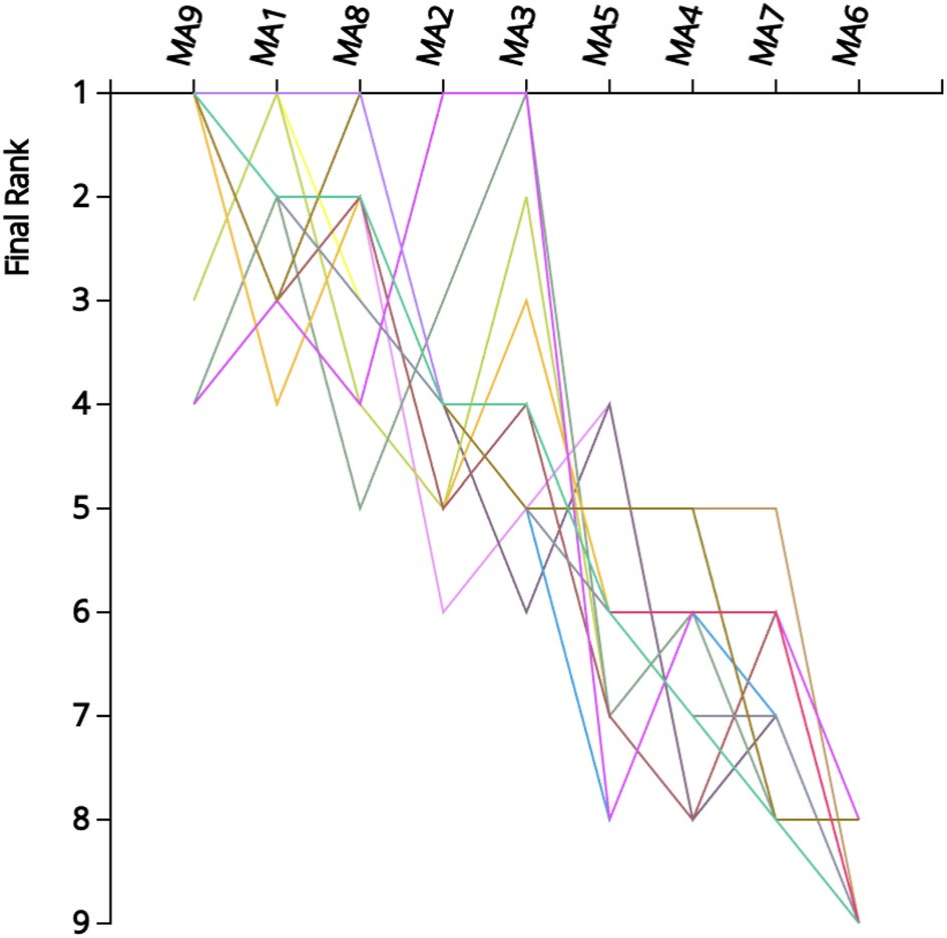
Comparisons of rankings over all normalization/aggregation combinations.

Figure 2 presents the comparison of rankings obtained by each location over all normalization/aggregation combinations. From these comparisons, it is evident that locations (MA4 ÷ MA7) never achieve a rank higher than four. This is the kind of observation that leads the negotiation team to consider dropping from the next iteration the locations that appear to not have a chance to rank at the top. It is a tempting consideration, as it will facilitate the deliberations by focusing on fewer solutions and thus will reduce the time needed to reach a final decision.

**Figure 3:**
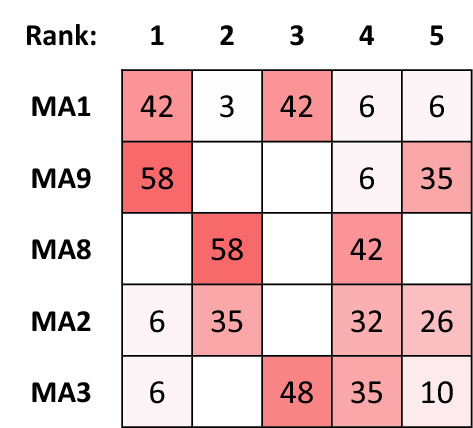
Ranking Strength over all normalization/aggregation combinations (5 alternatives).

**Figure 4:**
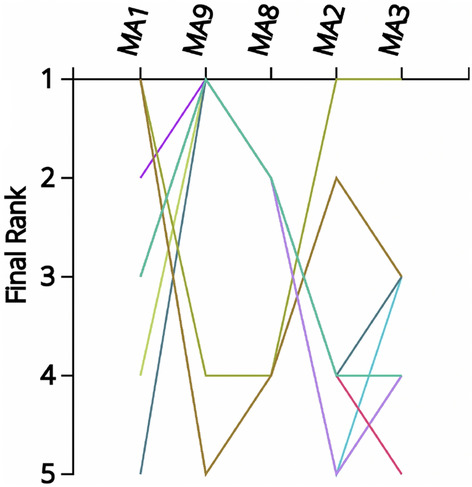
Comparisons of rankings over all normalization/aggregation combinations (5 alternatives).

If the negotiation team were to succumb to the temptation and re-structure the problem with only the five choices (MA1, MA2, MA3, MA8 and MA9) the results produced with the MCDA Index Tool will appear as in Figs. 3 and 4. The rankings have transformed dramatically and in fact location MA1 might be preferable over MA9 while the uncertainty of the choice has also increased significantly.

**Figure 5:**
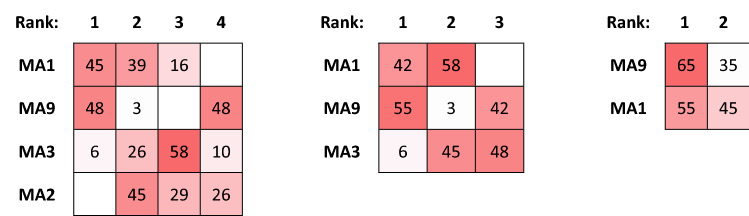
Progressive Ranking Strength through elimination.

This is the dreaded Rank Reversal (RR) paradox that plagues most MCDA techniques and presents a unique challenge to real-world problems^28^. Due to the RR paradox, the results could be different depending on included alternatives. For instance, adopting a strategy where in each step the alternative(s) with no first ranks are dropped and the process is repeated for the remaining ones. The results of this -admittedly arbitrary- elimination strategy are highlighted in Fig. 5. Clearly, the order of choice shifts from MA9 to MA1 and returns to the original state only when the two alternatives compete head-on.

There is a school of thought in MCDA that recognizes that the head-on comparison of MA9 and MA1 is a more reliable indicator of the preferred solution and proposes to compare *all* the alternatives directly one to one^29^. The Condorcet method, with origins in social choice theory, purportedly prevents distortions by making the relative position of two alternatives independent of their positions relative to any other^30^. For the example at hand, comparing the 9 alternatives head-on requires the MCDA analysis of 36 pairs. Table 2 summarizes the results obtained through this approach with the value of each cell indicating the ratio of the 1^st^ ranks achieved by the column-alternative to the 1^st^ ranks achieved by the corresponding row-alternative. If the number is more than 1, the column-alternative wins over the row-alternative; if it is less than 1, the row-alternative wins; and if it is exactly 1 there is a tie.

**Table 2:** Condorcet comparison of the alternatives.

	MA1	MA2	MA3	MA4	MA5	MA6	MA7	MA8	MA9
MA1		0.48	0.13	0.10	0.10	0.10	0.17	0.74	1.19
MA2	2.07		1.37	0.47	0.19	0.17	0.16	1.45	1.45
MA3	7.69	0.73		0.50	0.10	0.10	0.10	1.45	1.45
MA4	10	2.14	2		0.67	0.20	0.21	1.45	1.45
MA5	10	5.26	10	1.5		0.10	0.17	1.12	1.12
MA6	10	5.87	10	5.11	10		1.00	1.45	1.45
MA7	5.87	6.25	10	4.73	5.87	1.00		1.45	1.45
MA8	1.35	0.69	0.69	0.69	0.89	0.69	0.69		1.45
MA9	0.84	0.69	0.69	0.69	0.89	0.69	0.69	0.69	
WINS:	7	4	5	3	2			6	8
RANK:	2nd	5th	4th	6th	7th	8th	8th	3rd	1st

The Condorcet method appears to restore the approximate order obtained through the MCDA method of all the 9 alternatives examined together in Fig. 1, there are still interesting rank reversals. For instance, it restores MA8 as a contender, while this choice disappeared early in the elimination strategy.

## The Rank reversal conundrum and identifying the top choices

RR is a paradox because the rank order of alternatives can be changed when a current choice is eliminated from the set of alternatives or a new one is added. RR is a challenge because it undermines the credibility of ratings and rankings of MCDA and enhances the suspicions of stakeholders that rankings can be “manipulated” to advance pre-determined agendas.

Several different MCDA techniques have been proposed in recent years claiming to be rank-reversal-free^31^. The experience from the field though is that while these methods may successfully overcome RR problems, they are not completely reversal free. The most promising among them is the low-complexity Stable Preference Ordering Towards Ideal Solution (SPOTIS) approach^32^.

SPOTIS is based on the classical MCDM structure but requires additional information on the min and max bounds of score values for each criterion. These bounds along with the polarity of each criterion define the *ideal* best solution. For the offshore wind farm installation case study of the previous section, the ideal solution point is computed in Table 3. The SPOTIS method proceeds to compute the closeness of each alternative to the ideal point solution by utilizing a simple distance metric (E1) and normalizing it with respect to the distance between the min and max values for each criterion. This leads to a unitless average distance of each alternative from the multi-criteria ideal one^32^.

**Table 3:** The SPOTIS approach.

Bounds	AC1 (m)	AC2 (km)	AC3 (kV)	AC4 (km)	AC5 (Population)	AC6 (kWh/m^2^)	AC7 (km^2^)
min	50	25	66	50	119 987	1600	0.973
Max	100	50	400	70	686 969	1800	4.885
Polarity	Negative	Negative	Positive	Negative	Positive	Positive	Positive
Ideal Point	50	25	400	50	686 969	1800	4.885

Table 4 summarizes the average distances computed for the nine alternatives of the offshore wind farm installation and the resultant ranking. MA9 emerges clearly as the preferred solution, with MA8 and MA1 practically tied for second place.

**Table 4:** SPOTIS average distances from the ideal solution.

	Average Distance	RANK
MA9	0.317	1st
MA8	0.391	2nd
MA1	0.393	3rd
MA2	0.532	4th
MA3	0.588	5th
MA5	0.629	6th
MA4	0.687	7th
MA7	0.700	8th
MA6	0.960	9th

It is safe to assume that the negotiation team might consider dropping MA6 from the next iteration. Since the removal of MA6 does not change the bounds of the criteria (and this is indeed the case here) the average distances of the remaining alternatives remain the same and there is no rank reversal. If on the other hand, MA4 ÷ MA7 are removed as locations that appear to not have a chance to rank at the top the bounds, the ideal solution, and the average distances of the remaining five alternatives do change yet there is no rank reversal (Table 5).

**Table 5:** SPOTIS average distances from the ideal solution (5 alternatives).

	Average Distance	RANK
MA9	0.380	1st
MA8	0.532	2nd
MA1	0.600	3rd
MA2	0.793	4th
MA3	0.870	5th

While SPOTIS appears to be rank-reversal free for the case study at hand, it is not certain that the rankings it generates are superior to the MCDA techniques. Table 6 summarizes the alternative rankings computed to this point. It is apparent that while MA9, MA1 and MA8 are the top three contenders, with MA2 and MA3 following closely, the relative ranking differs depending upon the method used.

Clearly, there could have been many more MCDA techniques used since no one method can be considered the most appropriate for all situations. Multiple attempts in the literature to compare or benchmark methods against each other failed to produce results reproducible across a wide range of paradigms^33^. Beyond the fundamental technical aspects of each method, the use of MCDA requires a strong “craft” element^34^. Practitioners should be cognizant of the requirements, limitations, and peculiarities of each method in their field to use them effectively as well as of the fundamental observation that using a particular MCDA method can and does significantly influence the outcome.

**Table 6:**
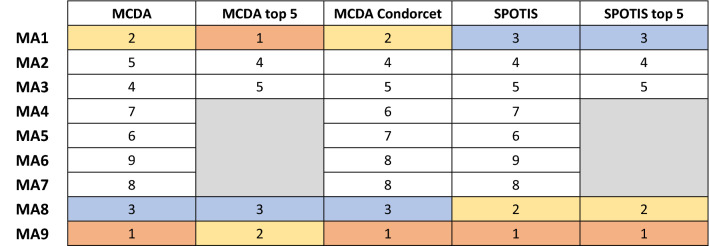
Summary rankings of MCDA, MCDA top 5, MCDA Condorcet, SPOTIS, and SPOTIS top 5.

In offshore construction, the combination of classical and Condorcet MCDA along with SPOTIS has been proven to be sufficient for the recognition of the frontrunners between the various alternative decision choices. (In the example considered, the 31 distinct combinations of normalization and aggregation methods assessed through the MCDA Index tool, the 36 one-on-one comparisons of the 9 alternative choices of the MCDA Condorcet method, and the results of the robust to rank-reversal technique of SPOTIS provide a sufficiently rich milieu to recognize the top choices.)

Identifying the frontrunners is essential for the second round of the planning process, where significant time, effort and funding will be spent on detailing the distinct characteristics of each alternative. Eliminating candidates during the first round is often a contentious issue with the stakeholders and the process should be such that it can withstand scrutiny. In real-world offshore construction, this often achieved with the use of an expert system.

**Figure 6:**
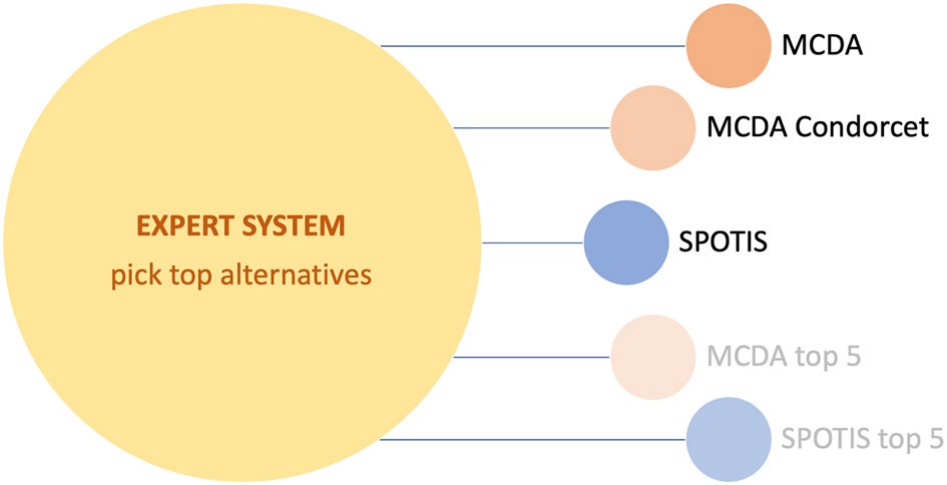
Expert System to identify the top alternatives.

Figure 6 illustrates succinctly the design of such an expert system that utilizes the rankings obtained via MCDA, MCDA Condorcet, SPOTIS (and, if there are many choices, MCDA top 5 and SPOTIS top 5) to pick the top alternatives.

The expert system is based on a knowledge platform that incorporates the expert knowledge and experience of the contractor in the field of the offshore construction. The knowledge base is continuously updated through a learning module, as new projects are added to the portfolio of the company and ongoing and completed projects are reviewed for a posteriori assessment of the choices made. The simple user interface requires only the input of the rankings of the various choices that emerged through the MCDA techniques applied. The inference engine operates on a set of relatively simple, yet proprietary, rules of the if–then type rather than through conventional procedural code.

The entire approach is based on fundamentally deterministic criteria, and this is a distinct limitation. (Standard sensitivity analysis can be used though to examine the extent to which changes in the weights and scores of the criteria influence the robustness of the rankings obtained through each technique). In offshore construction, real world criteria weights and scores are often assessed based on multiple conflicting information sources. To address such cases another approach is used based on fuzzy logic.

## Fuzzy logic and criteria clustering

The Characteristic Object METhod (COMET) has been proposed recently to address MCDA problems with fuzziness in the criteria. COMET achieved prominence because it has been proven to be immune to the RR paradox. This property is interesting, although it is unfair to compare it with classical MCDA methods as it requires additional information in the structuring of the decision problem^35,36^. COMET has been recognized in the offshore construction industry for: (i) its incorporation of fuzziness in the criteria; and (ii) its intuitive methodology for hierarchical clustering of the criteria. Each of these issues is addressed in turn.

Expert knowledge on the significance level of each of the criteria is used to convert its range of values to a triangular fuzzy number (m_1_, m_2_, m_3_) where m_1_ represents the smallest likely value, m_2_ the most probable value, and m_3_ the largest possible value of the fuzzy event. Table 7 indicates these characteristics values for the criteria of the running offshore wind farm installation case study. For criteria AC1, AC2, and AC4 the values of which are practically binary, only the two extremes are represented. The COMET method then proceeds by requiring an expert panel to score in terms of preference all pairwise combinations to create a rule base.

**Table 7:** Triangular fuzzy numbers for criteria AC1 ÷ AC7.

	AC1 (m)	AC2 (km)	AC3 (kV)	AC4 (km)	AC5 (Population)	AC6 (kWh/m^2^)	AC7 (km^2^)
m_1_	50	25	66	30	120 000	1600	1.5
m_2_	–	–	150	–	119 887	1700	3.0
m_3_	100	50	400	60	687 000	1800	4.5
Polarity	Negative	Negative	Positive	Negative	Positive	Positive	Positive

**Figure 7:**
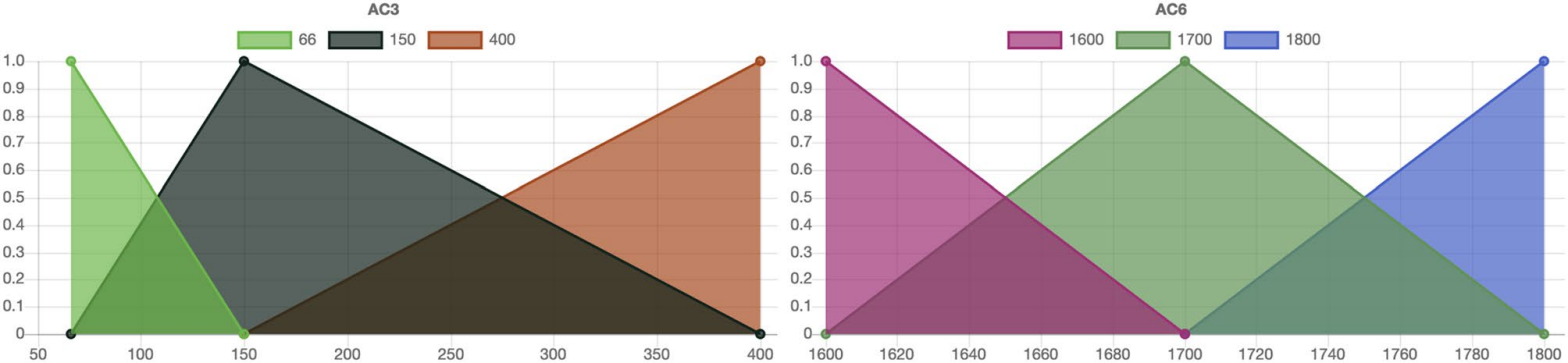
The set of three triangular fuzzy numbers for AC3 and AC6.

For the simplicity of the presentation, the assumption momentarily is of having just two criteria, say AC3 and AC6. Each combination of a distinct value of AC3 with a distinct value of AC6 is called a “characteristic object”, akin to a state vector in the (AC3, AC6) two-dimensional space. This in turn requires the expert valuation of whether, for instance, the combination (150 kV, 1600 kWh/m^2^) is preferred over (66 kV, 1700 kWh/m^2^). Both criteria are of the benefit type, hence higher values are preferable. If the expert panel consistently prefers the bigger incremental increase in AC3 over the less impressive step up in AC6, then the scoring of the 9 possible characteristic objects (CO) leads to the rule base in Table 8 and the triangular fuzzy numbers in Fig. 7. The rule base in Table 8 not only ranks the 9 possible pairs but also assigns a corresponding preference score between 0.0 (least desirable) and 1.0 (most desirable). The preference score can then be used to rank the alternative choices MA1 ÷ MA9 as in Table 9. MA8 and MA9 tie in first place and MA1, MA2 in second place while MA6 and MA7 are the least appealing alternatives.

**Table 8:** Complete rule base for criteria AC3 and AC6.

	AC3	AC6	*P*
CO1	66	1600	0.000
CO2	66	1700	0.125
CO3	66	1800	0.250
CO4	150	1600	0.375
CO5	150	1700	0.500
CO6	150	1800	0.625
CO7	400	1600	0.750
CO8	400	1700	0.875
CO9	400	1800	1.000
Polarity	Positive	Positive

**Table 9:** Scoring the alternative choices based on criteria AC3 and AC6 only.

	AC3	AC6	*P*	Rank
MA1	150	1800	0.625	2
MA2	150	1800	0.625	2
MA3	150	1700	0.500	3
MA4	66	1800	0.250	4
MA5	66	1700	0.125	5
MA6	66	1600	0.000	6
MA7	66	1600	0.000	6
MA8	400	1600	0.750	1
MA9	400	1600	0.750	1

The COMET technique is easy to implement and the online tool DSS COMET (www.comet.edu.pl) can automate the process. But its applicability is constrained by the fact that it is not practical for an expert panel to examine more than 2–3 criteria at a time. For instance, in the offshore wind farm installation case study there are three criteria with two options (AC1, AC2, AC4) and four criteria with three options (AC3, AC5, AC6, AC7). Processing the full problem with COMET will lead to N = 3^4^ ∙ 2^3^ = 648 characteristic objects, requiring ½N(N−1) = 209,628 pairwise comparisons. Above and beyond that fact that so many comparisons are exhausting for the expert panel, the human brain cannot make inferences with more than 3–4 items stored in the working memory. (Working memory is the active version of short-term memory related to the temporary storage and manipulation of information and its limited capacity is a central bottleneck of human cognition^37,38^).

This apparent “curse of dimensionality” has led the authors of the COMET technique to propose a practical alternative by decomposing the problem into smaller ones through clustering of similar criteria^39^. Creating a structure of decisional models interconnected with each other significantly reduces the number of pairwise comparisons needed as well as the cognitive load on the expert panel.

**Figure 8:**
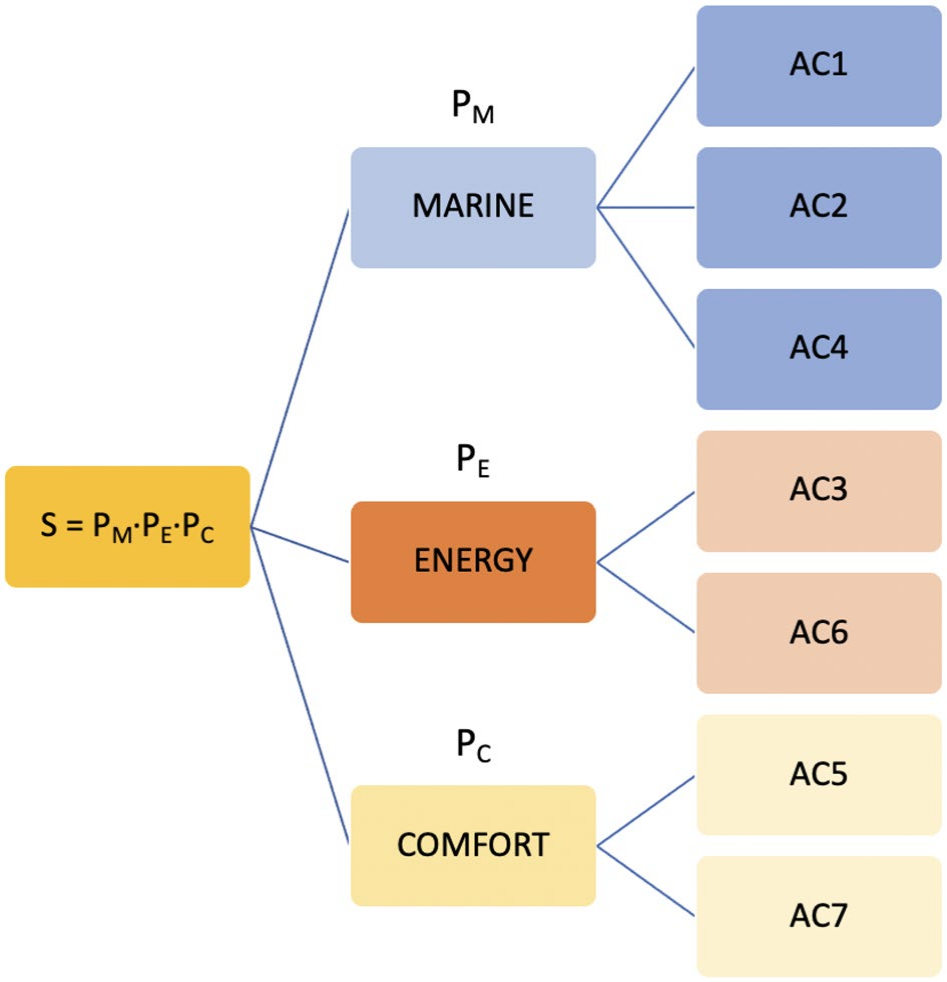
Hierarchical decomposition through criteria clustering and expert scoring.

In the running case study, it is plausible to group criteria AC1, AC2 and AC4 under a “*marine*” banner; AC3 and AC6 under an “*energy*” banner; and AC3 and AC7 under a “*comfort*” banner. Figure 8 depicts the hierarchical structure of decomposing the full problem into three smaller ones.

A separate score is computed for each of the three sub-problems and a final composite score is produced as the product of the scores every alternative receives from each sub-problem. This modified COMET approach leads to 3^4^ + 2^3^ = 89 characteristic objects and a total set of 324 + 28 = 352 pairwise comparisons. (Admittedly, these savings are achieved by not solving the complete problem and hence immunity to RR is no longer guaranteed.)

The “energy” criteria were already examined in Table 8 and Fig. 7 and the results in Table 9 are incorporated in column P_E_ of Table 10. For brevity, the results for the “marine” criteria and “comfort” criteria are also summarized in columns P_M_ and P_C_ respectively of Table 10.

**Table 10:** Scoring alternatives in the marine sub- problem.

	AC1	AC2	AC4	P_M_	AC3	AC6	P_E_	AC5	AC7	P_C_	*P*	Rank
MA1	100	25	30	0.571	150	1800	0.875	687	1.5	0.625	0.846	1
MA2	100	25	30	0.571	150	1800	0.875	120	1.5	0.000	0.462	3
MA3	100	25	30	0.571	150	1700	0.625	176	1.5	0.250	0.308	5
MA4	100	25	60	0.143	66	1800	0.500	176	1.5	0.250	0.077	8
MA5	100	50	30	0.289	66	1700	0.250	176	4.5	0.750	0.154	6
MA6	100	50	60	0.000	66	1600	0.000	176	1.5	0.250	0.000	9
MA7	50	25	60	0.714	66	1600	0.000	176	1.5	0.250	0.154	6
MA8	50	25	30	1.000	400	1600	0.375	176	1.5	0.250	0.462	3
MA9	50	25	30	1.000	400	1600	0.375	176	3.0	0.500	0.692	2

In the interest of reproducibility of the results, the expert scoring of the COs for the values of AC1, AC2, AC4 favors water depth over distance from shore or from port. Similarly, the expert scoring of the COs for the values of AC3, AC6 and AC5, AC7 favors the pairs that exhibit higher values by a bigger margin. (For example, the AC3/AC6 pair 400/1700 is preferred over the 66/1800 one.) In the interest of brevity, Table 10 summarizes directly the scores achieved by each alternative in the evaluation of each sub-problem as well as the composite score and ranking.

From the results of the various methods in Table 6, the option MA9 is the top choice, followed by MA1 and MA8. But the SPOTIS results in Table 10 indicate that MA1 is the top choice, followed by MA9 and MA8, with MA9 holding a distinct 22% advantage over MA1. An additional advantage of the COMET approach is that allows for straightforward assessment of the values of the criteria that may shift the rank position of a choice^36^.

## Intelligent MCDA methodology

It should be apparent at this point that MCDA can assist individuals and organizations to make better decisions. But the outcome cannot be the automatic result of an MCDA algorithm; it should always be a decision made by the stakeholders after an exhaustive review of all the data at hand. In the case of offshore construction, the complexity of the tasks involved and the need to engage diverse groups of experts and stakeholders in the process makes matters more difficult.

In offshore construction where time is of essence, experienced contractors make sure that there is real participation and deliberation in the application of MCDA to reduce unnecessary iterations. Yet, iterations *are* necessary to reach an acceptable and satisfactory outcome.

**Figure 9:**
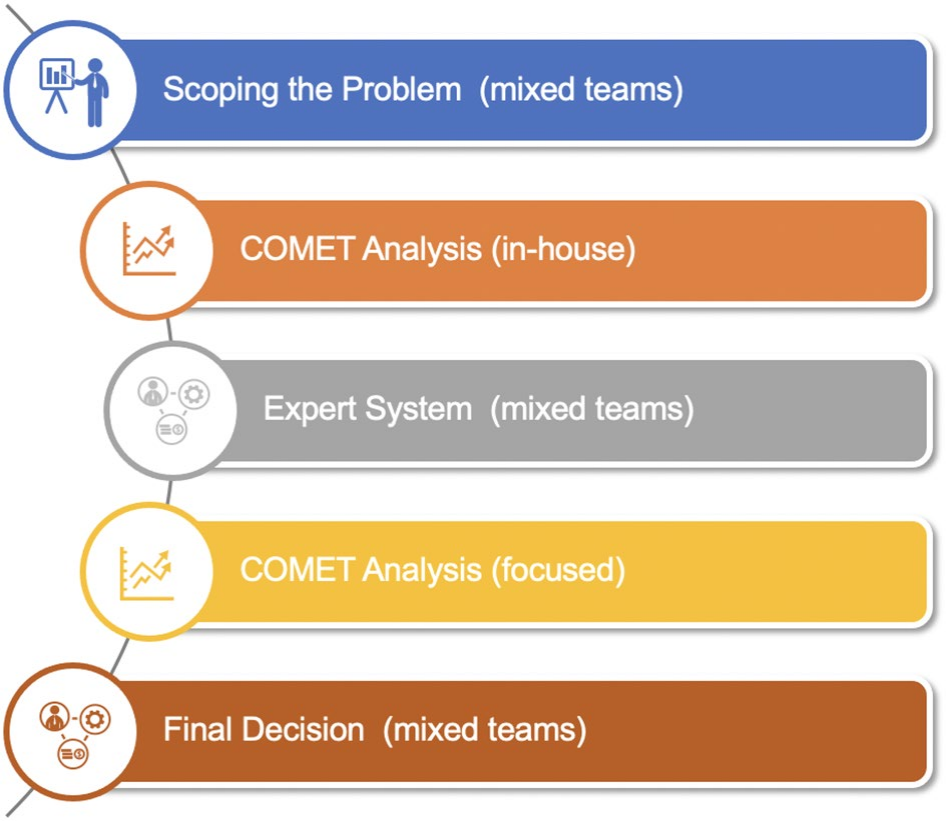
An intelligent framework for MCDA use in offshore construction.

Figure 9 captures the proposed intelligent framework base on the mathematical foundations of MCDA and the real-world experience in offshore construction. The process starts with the phase of scoping the problem, identifying a basic set of choices, and establishing the criteria for the decision-making process. This phase involves mixed teams from the contractor and the stakeholders and, possibly, outside experts. Once the initial scope of the problem is set, the contractor team performs an in-house COMET analysis to comprehend better the characteristics of the choices involved, to identify potential rankings and to develop a sense of the impact of the various criteria.

The third phase involves the use of the expert system defined in Fig. 7, with mixed teams for the contractor and the stakeholders. The desired outcome of this phase is a smaller set of alternatives and fewer criteria -but it is quite possible that the process might need to re-start with new alternatives added to the mix as the continuous exposure of the stakeholders to the issues involved might change their view on the scope of the problem.

If a smaller, more realistic set of choices emerges from the expert system phase the contractor team performs another, more focused, COMET analysis in-house to inform the final decision phase. In this final phase, participation extends beyond information dissemination to include active dialogue and debate between the participants. It is again possible that the scope of the problem might be further modified in lieu of a final decision and the process will have to restart^38^. Real-world experience demonstrates that this is unlikely to happen if the previous phases have been carefully choreographed.

## Conclusions

Impact assessment, the evidence-based prospective impact analysis part of the planning stage of any development project, is subject to regulatory oversight providing for public engagement, reconciliation, and partnership in the public interest during the design phase. Indeed, impact assessment of large-scale projects involves a plethora of technical, economic, social, and environmental factors that must be assessed along with the expectations of the stakeholders of each project.

Molding the multiple perspectives of diverse stakeholders into a single collective choice is a key challenge in impact assessment and MCDA is the de facto methodology used to rank decision options based on a predetermined set of evaluation criteria. Different MCDA techniques, however, may lead to different decisions when applied to the same problem, while different sets of criteria and weights may rank choices differently even when the same method is applied. This is a cause of concern, and even acrimony, amongst the stakeholders, often leading to protracted periods to protracted negotiations and delaying construction.

The objective of this paper was to ameliorate the effects of the inherent subjectivity in MCDA techniques and to develop a consensus amongst the stakeholders in a data-driven setting. This was accomplished not by devising a new MCDA technique but, rather, through a novel IDSS employing *existing* methods from the MCDA toolbox and implemented via web-based software. The design of the system is informed *both* by theoretical MCDA (and COMET in particular) and by field experience.

While the intelligent methodology presented in this paper has been detailed through the running example of a case study from offshore construction, the proposed approach is directly applicable to all large-scale projects requiring impact assessment throughout their design phase. Indeed, real-world offshore construction is representative of the field of large-scale projects where a plethora of technical, economic, social, and environmental factors collude to create a morass of complex issues and expectations that are difficult to assess in a uniform canvas.

It would be natural at this point to offer to fully automate the process as an extension of current research. It is the strong conviction of the authors, though, that a desirable outcome cannot be the product of an automated process. The criteria used as well as their respective weights are products of expert opinion and cannot be fully captured by an expert system. The deliberative nature of the proposed framework, while cumbersome, is essential to form consensus especially when the technical, economic, and regulatory issues involved created an often-fuzzy decision tableau.

## Data Availability

The original case study data have been published in^26^, publicly available at https://www.mdpi.com/2077-1312/10/2/224. All other data generated or analyzed during this study are included in the present manuscript.
